# Carnosine Treatment for Gulf War Illness: A Randomized Controlled Trial

**DOI:** 10.5539/gjhs.v5n3p69

**Published:** 2013-02-04

**Authors:** James N. Baraniuk, Suliman El-Amin, Rebecca Corey, Rakib U. Rayhan, Christian R. Timbol

**Affiliations:** 1 Divison of Rheumatology, Immunology and Allergy, Georgetown University, Washington DC, USA

**Keywords:** clinical trial, Chronic Fatigue Syndrome, homocarnosine, digit symbol substitution test

## Abstract

About 25% of 1990-1991 Persian Gulf War veterans experience disabling fatigue, widespread pain, and cognitive dysfunction termed Gulf War illness (GWI) or Chronic Multisymptom Illness (CMI). A leading theory proposes that wartime exposures initiated prolonged production of reactive oxygen species (ROS) and central nervous system injury. The endogenous antioxidant L-carnosine (β-alanyl-L-histidine) is a potential treatment since it is a free radical scavenger in nervous tissue. To determine if nutritional supplementation with L-carnosine would significantly improve pain, cognition and fatigue in GWI, a randomized double blind placebo controlled 12 week dose escalation study involving 25 GWI subjects was employed. L-carnosine was given as 500, 1000, and 1500 mg increasing at 4 week intervals. Outcomes included subjective fatigue, pain and psychosocial questionnaires, and instantaneous fatigue and activity levels recorded by ActiWatch Score devices. Cognitive function was evaluated by WAIS-R digit symbol substitution test. Carnosine had 2 potentially beneficial effects: WAIS-R scores increased significantly, and there was a decrease in diarrhea associated with irritable bowel syndrome. No other significant incremental changes were found. Therefore, 12 weeks of carnosine (1500 mg) may have beneficial cognitive effects in GWI. Fatigue, pain, hyperalgesia, activity and other outcomes were resistant to treatment.

## 1. Introduction

Gulf War illness (GWI) is a multisymptom disorder affecting an estimated 25% of the 700,000 military veterans of the First Persian Gulf War (1990-91) (Fukuda et al., 1998; Kang, Li, Mahan, Eisen, & Engel, 2009; Research Advisory Committee on Gulf War Veterans’ Illnesses [RAC-GWVI], 2008). GWI veterans have a wide spectrum of “functional” complaints including disabling fatigue, diffuse musculoskeletal pain, gastrointestinal dysfunction, sleep abnormalities, and cognitive impairments (Spencer et al. 1998; McCauley et al. 2002; Steele 2000). Neurological dysfunction may be a common pathophysiological mechanism. This would be consistent with the hypothesis that war time exposures to combinations of low levels of sarin and other nerve gas agents, the acetylcholinesterase inhibitor pyridostigmine bromide, organophosphate and other pesticides, particulate and volatile organic compounds in smoke from oil-well fires, depleted uranium, immune activation by simultaneous administration of multiple vaccines and their adjuvants, and stressors of deployment to a hot, dry, dehydrating environment may have acted synergistically to initiate central nervous system injury (RAC-GWVI, 2008; Steele, Sastre, Gerkovich, & Cook, 2012; Glass et al., 2006; Hernández et al., 2012; Harley et al., 2011; Block et al., 2004). Stressors combined with pyridostigmine bromide, N, N-diethyl-m-toluamide (DEET), and permethrin can disrupt the blood brain barrier and cause neuron death in animal models (Abdel-Rahman, Shetty, & Abou-Donia, 2002). Reactive oxidant species (ROS) may mediate the neuronal damage (Golomb, 2008; Li, Shou, Borowitz, & Isom, 2001).

If ROS production is a continued component of GWI neuropathology, then brain antioxidants may have a critical protective role. Differences in production or turnover of endogenous or supplemental antioxidants may be one factor that discriminates between veterans who have developed GWI and those without the syndrome. L-Homocarnosine (γ-aminobutyric acid – L-histidine) is the premier antioxidant dipeptide in the brain. Unfortunately, this chemical is not available for clinical use. L-Carnosine (β-alanine-L-histidine) is a closely related dipeptide that may scavenge ROS in the central nervous system (Boldyrev, Dupin, Bunin, Babizhaey, & Severin, 1987; Kohen, Yamamoto, Cundy, & Ames, 1988) and act as an antioxidant and buffering ion in muscle and possibly other peripheral tissues (Abe, 2000; Holliday & McFarland, 2000). Carnosine may promote brain health because autistic children taking 800 mg a day demonstrated significant cognitive improvements (Chez et al., 2002). This suggested that carnosine may cross the blood brain barrier.

The primary aims of this pilot Phase I/II study were to determine if carnosine improved activity and instantaneous fatigue scores (ActiWatch Score accelerometer device, MiniMiter, Bend, OR) (Gironda, Lloyd, & Clark, 2007; Kim et al., 2010), Chronic Fatigue Syndrome (CFS) Severity Score (Baraniuk, Clauw, & Gaumond, 1998; Baraniuk, Naranch, Maibach, & Clauw, 2000) pain and tenderness (dolorimetry) (Naranch et al., 2002) and quality-of-life domains (Sherbourne, 1995) compared to placebo in GWI subjects. Psychometric and cognitive measures were secondary outcomes.

## 2. Materials and Methods

### 2.1 Study Design

This randomized double blind placebo controlled pilot study of carnosine (500 mg capsules, Village Green Apothecary, Bethesda, MD; Food and Drug Administration Investigational New Drug FDA IND # 101,700) versus indistinguishable microcrystalline cellulose capsules was tested as add-on therapy in GWI. Dose escalation started with 500 mg once daily, and increased to 500 mg twice daily, and then 1,000 mg in the morning and 500 mg in the evening (1,500 mg/day) at 4 week intervals (ClinicalTrials.gov identifier NCT00810368). Sets of 4 × 100 capsule bottles were prepared and blocks with 3 placebo and 3 carnosine sets randomly mixed. Each set was assigned a sequential 6 digit alpha-numerical identification code so study drug could be dispensed by research pharmacists not involved in blinding the study. After the week 12 visit, the 3 capsules per day were tapered by 1 per week until discontinued. The study was approved by the Georgetown University Institutional Review Board (ID # 2008-068). Data analysis, recruitment, protocol implementation, and patient participation all occurred at Georgetown University Medical Center and Hospital in Washington, D.C. of the United States.

The planned sample size of n=13 subjects completing each arm was based on individual incremental improvements (Δ) in the primary outcomes of: (i) CFS Severity Score (Δ ≥ 5 / 32) (Baraniuk et al., 1998; Baraniuk et al., 2000a; Baraniuk, Naranch, Maibach, & Clauw, 2000b); (ii) instantaneous fatigue (Δ ≥ 2 using the 0 to 10 point ordinal scale) (Kim et al., 2010); (iii) activity levels (Δ ≥ 20%) using ActiWatch Score accelerometer devices (Gironda et al., 2007); and (iv) increased accuracy of 4 / 35 letters on 2-back working memory tasks performed during fMRI testing (Owen, 2005; Caseras et al., 2006; Altman, 1991; Altman, 1982; Cohen, 1988) (The 2-back tests and fMRI results will be reported elsewhere). Secondary outcomes are described below. Stringent, conservative criteria for significant treatment responses required: (a) balanced groups with comparable week 0 values, (b) significant improvement with carnosine but not placebo by 2-tailed paired Student’s t-tests (p ≤ 0.05), and (c) significant differences between the 2 treatment responses at week 12 (p ≤ 0.05 by 2-tailed unpaired Student’s t-tests). Fisher’s Exact test was used when required. Data were reported as means and incremental changes (Δ) between weeks 0 and 12 ± standard deviations (SD).

### 2.2 Subject Recruitment

Subjects were recruited by on-line and newspaper advertisements, and other contacts. Telephone informed consent was obtained to determine eligibility and contact information. GWI subjects were eligible if they met the 1998 CDC criteria (Fukuda et al., 1998; RAC-GWVI, 2008) or had been diagnosed with chronic fatigue syndrome after Persian Gulf deployment (Gray, Reed, Kaiser, Smith, & Gastanaga, 2002). Exclusions included active military personnel, pregnant women, children, persons suffering from major chronic illnesses or psychiatric disorders, and persons with metal implants or other exclusions from fMRI testing (Fukuda et al., 1998; Kang et al., 2009; RAC-GWVI, 2008; Spencer et al., 1998; McCauley et al., 2002; Steele, 2000).

### 2.3 Study Questionnaires

The CFS Severity score allowed subjects to score the cardinal features of fatigue, cognition, sore throat, lymph nodes, myalgia, arthralgia, sleep and exertional exhaustion (Fukuda et al., 1994; Reeves et al., 2003; Reeves et al., 2005) as none (0), trivial (1), mild (2), moderate (3), or severe (4) (Baraniuk et al., 1998; Baraniuk et al., 2000a; Naranch et al., 2002; Wasserfallen, Gold, Schulman, & Baraniuk, 1999). The maximum sum for this severity score was 32. Medical Outcome Survey Short Form 36 (SF-36) domains were assayed for changes in quality of life (Ware & Sherbourne, 1995). Additional secondary symptom and psychometric questionnaires were completed to corroborate and expand data on GWI symptom co-variance and overlap with other syndromes (see Supplementary online material, SOM). The instruments were assessed for magnitudes, standard deviations, and reproducibility between weeks 0 and 12 to determine which may be of value for future studies. Subjects could complete the questionnaires on paper or our on-line, Google-based system. Each subject received a personal log-in, password and confidential identification code to access and score the questionnaires (Zheng et al., 2011a; Zheng et al., 2011b). No personal identifying information was collected over the internet. All data were stored in confidential fashion on a password protected computer.

### 2.4 Study Visits

If eligible, subjects came to the Clinical Research Unit, Georgetown University Hospital, Washington, D.C., for their screening visit. After reviewing the study protocol and participant responsibilities, subjects gave written informed consent for participation. Mental competence was assured by Mini-Mental Status examination (Folstein, Folstein, & McHugh, 1975). History was confirmed using a scripted interview that included diagnostics for CFS (Fukuda et al., 1994), fibromyalgia (Wolfe et al., 1990; Wolfe et al., 2010), myalgic encephalomyelitis (ME) (Carruthers et al., 2011), irritable bowel syndrome (Lea, Hopkins, Hastleton, Houghton, & Whorwell, 2004; Kurland, Coyle, Winkler, & Zable, 2006) and migraine (Headache Classification Subcommittee of the International Headache Society, 2004; Ravindran, Zheng, Timbol, Merck, & Baraniuk, 2011).

Subjects had physical examination, counting of manual tender points (Wolfe et al., 1990) and dolorimetry (Naranch et al., 2002). Screening blood work was drawn for HIV, pregnancy, blood counts, transaminases, and markers of inflammation. Cognitive function was assessed by Trail Making tests A and B (Corrigan & Hinkeldey, 1987) and digit symbol substitution test (Joy, Fein, Kaplan, & Freddman, 2000). Subjects toured the Center for Function and Molecular Imaging (CFMI) and practiced 0- and 2-back testing until they felt competent. They returned later for their fMRI with cognitive testing.

Subjects wore ActiWatch-Score wrist devices (Gironda et al., 2007) for the first 2 weeks of the study, and weeks 12 to 14. In preliminary tests, it was difficult to discriminate between periods of rest and sleep related to chronic fatigue and sedentary work (e.g. computer) performed by control subjects. Diaries were consulted to confirm periods of rest and sleep. Average accelerometer values for each 1 hour epoch during sedentary periods were < 40 units (mean + 2 standard deviations). This threshold distinguished “rest” from “active” periods. Records were reviewed for times when the watches were removed (zero activity). The percent of active epochs (% Active Time) during the entire time the devices were worn was used as an outcome variable. The average activity levels during active periods were also calculated on the assumption that treatment may be associated with more vigorous or prolonged movement. The Score alarm buzzed randomly at approximately 1 hour intervals to remind subjects to grade their instantaneous fatigue on a scale of 0 (no fatigue) to 10 (worst fatigue in life) (Kim et al., 2001). The average fatigue score for each day and the standard deviations and coefficients of variation were calculated to assess the magnitude of daily fluctuations in this symptom, and the trends for fatigue within each 2 week period.

Contact was maintained during the study by telephone and e-mail. Subjects who developed adverse events or discontinued participation were quizzed for their rationale. Symptoms, laboratories, and questionnaires were assessed at the week 6 safety visit. The week 12 visit followed the same format as week 0.

## 3. Results

### 3.1 Subjects and Adverse Events

Between February 2008 and November 2011 a total of 358 subjects were contacted about participation. Two hundred were interested and began the verbal screening process. Thirty-four subjects were enrolled and randomized. Nine subjects did not complete the study. Two were terminated. One developed an exacerbation of their typical severe musculoskeletal costochondritic chest pain, but was referred by their primary care physician to a cardiologist (Grade 1 adverse event, not related). Another was found to have elevated globulins at week 6. This participant did not return for follow-up or respond to any inquiries, and was lost to contact (Grade 2 adverse event, not related). Three subjects stopped participation within the first 2 weeks of the study and did not reply to any inquiries.

Three subjects did not want to continue in the study after they reached the 6 week mark because they were frustrated by the absence of any improvements. One moved away after 8 weeks. One subject was found to have an asymptomatic elevation of alanine-serine transaminase plus interval increase in CRP (remained in normal range). As per protocol, study drug was discontinued for 2 weeks. Hepatitis A, B and C serologies were negative, and tests normalized. Study drug was restarted and no alterations in symptoms or blood tests developed. This was considered a Grade 1 adverse event not related to study drug. Seven of these responses were in carnosine subjects, with two that dropped out after 6 weeks being on placebo. There were no demographic differences between the carnosine and placebo treated subjects who were enrolled or who completed the study (Table 1).

**Table 1:** Demographics

Treatment Group	Carnosine	Placebo
**Total (N)**	12	13
**Adverse Effect**	0	1
**Mean Age (± SD)**	51.8 ± 11.6	47.2 ± 8.7
**Male N**	8 (67%)	9 (64%)
**Female N**	4 (33%)	5 (36%)
**BMI (± SD) kg/m^2^**	31.5 ± 4.3	30.3 ± 5.7

As shown in Table 1, subjects who completed the twelve week protocol. BMI=Body Mass Index, SD=Standard Deviation. 17 out of 25 (68%) participants were male.

### 3.2 Cognition

The WAIS-R digit symbol substitution test (Joy et al., 2000) was the only instrument to show a significant carnosine-related change in score. The carnosine treatment group scores improved significantly between week 0 and week 12 (p=0.0018 uncorrected, 0.046 corrected) while the placebo group showed no change (Figure 1). The week 12 – week 0 incremental changes were significantly different between the carnosine and placebo groups (p=0.013, unpaired t-test, uncorrected). The carnosine group had arithmetically lower scores at week 0 than the placebo group (p = 0.089), while average scores were equal at week 12 (p = 0.66). This may have indicated that the week 0 scores were unbalanced between the 2 treatment groups. Although highly suggestive, our conservative criteria did not allow this to be considered conclusive evidence for a beneficial carnosine treatment effect on cognition. Post-hoc analysis indicated that sample sizes of 100 per group would show a significant benefit at 12 weeks (p < 0.05 corrected, power > 80%) if the week 0 average scores were equal, and the incremental increases remained 10.8 for carnosine and 2.7 for placebo. Trail Making tests were not different between weeks or groups.

**Figure 1:**
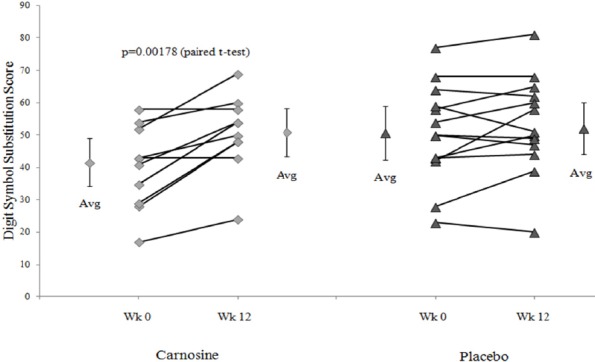
WAIS-R Digit Symbol Substitution scores for wk12 - wk0 (Δ)

The incremental change between weeks 12 and 0 (Δ) was significant by paired t-tests for the carnosine group (diamonds, average ± 95% CI on the left and right) (p=0.0018 uncorrected) but not for the placebo group (triangles, average ± 95% CI on the left and right). The magnitude of the changes was larger for the carnosine than placebo treatment (p=0.013 uncorrrected). Week 0 scores were higher for placebo (triangles) than carnosine (diamonds) (p=0.089), but were nearly identical at week 12.

### 3.3 Activity and Instantaneous Fatigue

Compliance was variable in both treatment groups. Chief complaints were poor comfort and loudness of the alarm prompt. The % active time was equivalent for the 2 groups and at both time points (Figure 2). Average activity levels were not affected. Instantaneous fatigue ratings showed surprisingly wide variations between GWI individuals (Figure 3). Fatigue levels were not altered in either treatment group and throughout both observation periods since the standard deviations for each subject were small and not related to fatigue severity. This emphasized the need to examine incremental changes as treatment outcomes rather than group differences.

**Figure 2:**
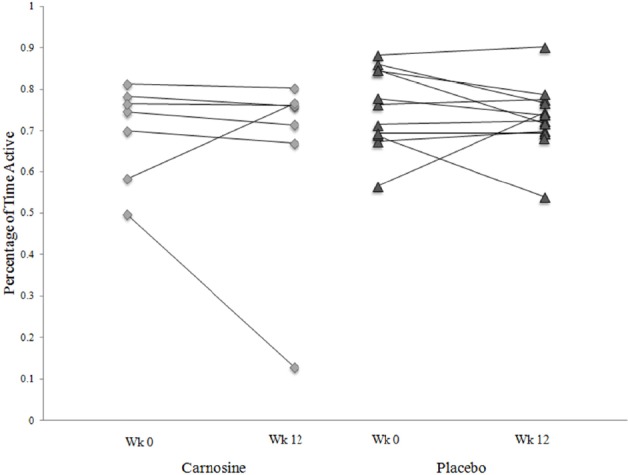
Percent of time active The percent of time active (accelerometer scores averaged each hour ≥ 40 units) was comparable for the carnosine (diamonds) and placebo (triangles) groups during the initial and final recording periods. The outlier in the carnosine group did not wear the watch for most of the second period.

**Figure 3:**
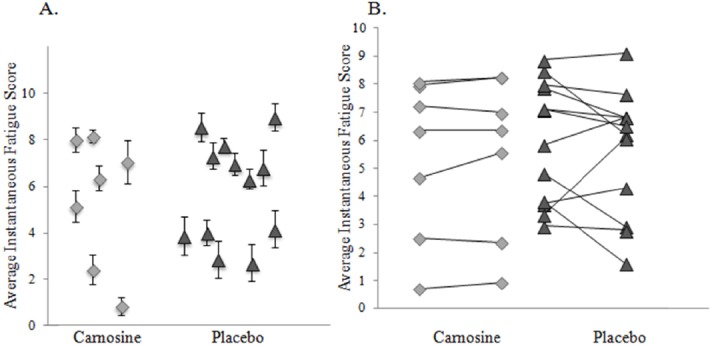
Instantaneous Fatigue Scores **A.** The average instantaneous fatigue scores and 95% confidence intervals from all measurements were shown for all subjects. Fatigue was a stable variable without large excursions (small CI’s) within individuals during the recording periods. **B.** There was a wide range of instantaneous fatigue scores within the carnosine (diamonds) and placebo (triangles) groups. Individual responses did not change between the two recording periods. There were no treatment effects.

### 3.4 Questionnaires

None of the variables of the CFS Severity Score were altered by treatments, nor were scores different between treatment groups (see SOM). These complaints were robust, reproducible, and resistant to current therapies. The results corroborated the instantaneous fatigue scores. SF-36 domain scores ranged from 65 for Physical Functioning to 11 for Vitality. This indicated significant, persistent disability in GWI over the 3 months of this study. There were no significant incremental differences for any of the questionnaires or their domain scores (SOM table).

### 3.5 Irritable Bowel Syndrome

Gastrointestinal complaints were assessed using Rome II criteria (Lea et al., 2004). At week 0, the carnosine group had 50% mixed, 0% constipation and 20% diarrhea predominant, and 30% with no irritable bowel syndrome. After 12 weeks, the proportions changed significantly to 14% mixed, 29% constipation, 14% diarrhea and 43% no irritable bowel syndrome (p = 0.019 by Fisher’s Exact test). The placebo group had no changes (38% mixed, 10% constipation, 19% diarrhea, 33% no irritable bowel syndrome). Carnosine reduced stool frequency and watery consistency while placebo did not.

### 3.6 Dolorimetry

Average pain thresholds were not altered by carnosine (Δ = -0.18 kg (SD 0.34)) or placebo (Δ = -0.67 ± 1.73 kg) treatments. The average of all measurements for GWI (3.32 ± 2.28 kg) was significantly lower than healthy controls from a parallel study (5.11 ± 2.77 kg; n=14; p = 0.035 by 2-tailed unpaired Student’s t-test). Pain thresholds were stable over the 3 month period, and may be a robust outcome measure to assess treatment effects on the neural plasticity associated with pain perception and as a long-term indicator of nociception, hyperalgesia, and central sensitization (Latremoliere & Woolf, 2009; Pietrobon, 2005; Baron, 2006).

## 4. Discussion

Carnosine treatment was associated with increased digit symbol substitution scores suggesting a cognitive benefit. This test has been associated with activation of the fronto-parietal brain network (Usui et al., 2009; Geake & Hansen, 2010). In contrast, there were no changes for Trail Making tests A and B that may assess inter-hemispheric cognitive processing (Corrigan & Hinkeldey, 1987). If so, carnosine may have selective trophic or protective effects in discrete brain regions.

Carnosine treatment was also associated with reduced stool frequency indicating an improvement in diarrhea. Other questionnaire outcomes including the primary aims were not altered by either carnosine or placebo treatments. Subjects’ levels of complaints were remarkably reproducible between week 0 and week 12. The symptoms caused significant disability based on SF-36 quality-of-life scores.

The absence of a placebo effect was important because the expectations of improvement did not translate into symptomatic improvement. This steadfast reporting demonstrated that GWI veterans were a highly reliable group who report their symptoms in a reproducible fashion. As a result, beneficial treatment effects in other studies are likely to be true results. The study drug was add-on therapy. The chronic severity of complaints suggests that current treatments had limited therapeutic benefits.

An extensive data set (SOM) was collected for (a) comparisons to CFS subjects, (b) potential characterization of CMI subtypes, (c) post hoc hypothesis generation, (d) selection of appropriate questionnaires for future studies, and (e) sample size calculations. Questionnaires assessed fatigue, pain, and interoceptive complaints such as rhinitis, dyspnea, irritable bladder and irritable bowel syndromes, and perceived responses to irritant exposures.

The cognitive improvement suggested by this study may be analogous to changes in autism. Carnosine given as 800 mg/d for 8 weeks in double blind, placebo controlled fashion to autistic children significantly improved behavior, socialization, communication and total scores on the Gilliam Autism Rating Scale (Chez et al., 2002). The mechanism(s) may include antioxidant actions via the terminal free amino group of β-alanine, anticonvulsant properties akin to GABA-ergic agents, chelation of copper and zinc at GABA receptor sites, proton buffering, antioxidant and anti-ischemic protection (Ozonoff, Pennington, & Rogers, 1991; Trombly, Horning, & Blakemore, 1998; Stvolinsky & Dobrota, 2000).

Carnosine improves athletic capabilities, probably due to antioxidant effects or buffering capacity in muscle (Abe, 2000; Holliday & McFarland, 2000). Physiological stressors acutely increased avian muscle carnosine by 10-fold, but had no effect on brain levels (Manhiani et al., 2011). Similar stressor effects have not been studied in man.

Carnosine is transported across cell membranes by the proton-coupled PepT2 oligopeptide transporter (Jappar, Hu, Keep, & Smith, 2009). About 14% is secreted intact into urine within 5 hours (Gardner, Illingworth, Kelleher, & Wood, 1991). The remainder may be cleaved by the enzyme carnosine dipeptidase 1 and 2 (CNDP1 and CNDP2) (Teufel et al., 2003) in the blood or kidney, or transported into liver, muscle or potentially across the blood brain barrier (Peters et al., 2011).

Carnosine digestion by CNDP1 and CNDP2 releases β-alanine and histidine. The latter can chelate metals and scavenge singlet oxygen during ischemia (Abe, 2000; Holliday & McFarland, 2000; Lee et al., 1999). Increased histidine absorption into the brain was hypothesized to trigger an increase in brain homocarnosine synthesis by the rate limiting enzyme ATP-grasp domain-containing protein 1 (ATPGD1) in astroglial cells (Drozak, Veiga-da-Cunha, Vertommen, Stroobant, & Van Schaftingen, 2010). Lumbar punctures and measurements of histidine, carnosine and homocarnosine in cerebrospinal fluid would be required to confirm this hypothesis.

β-Alanine is the rate limiting substrate for carnosine synthesis by ATPGD1 in human and equine muscle, since histidine supplements do not boost carnosine levels (Dunnett & Harris, 1999). β-Alanine supplementation improved performance in anaerobic muscle testing (Hoffman et al., 2008). β-Alanine may delay the onset of neuromuscular fatigue in intensively exercising athletes (Artioli, Gualano, Smith, Stout, & Lancha 2010). Similar effects may not have been evident in our GWI subjects since they were not enrolled in structured aerobic or anaerobic training programs or tested for aerobic exercise capacity as part of this pilot study. High doses of β-alanine (3 and 6 g/d) improved oxygen utilization during high intensity exercise training in elite athletes (Smith et al., 2009; Walter, Smith, Kendall, Stout, & Cramer, 2010), although lower doses do not have effects on VO_2MAX_ during aerobic testing.

Oral doses greater than 800 mg of β-alanine may induce paresthesias (Artioli et al., 2010). This is of relevance to our study since β-alanine (Shinohara et al., 2004) and ATP may bind Mrgprd receptors of murine dorsal root ganglion neurons that mediate cutaneous mechanical nociception (Cavanaugh et al., 2009). We found a higher drop-out rate in carnosine than placebo treated GWI veterans. They did not report any changes in cutaneous perceptions when questioned, but a subliminal effect cannot be ruled out in those subjects who were lost to contact. Perceptions of enteric interoceptive sensations may have developed based on changes in gastrointestinal complaints. At week 0, half of the carnosine group reported irritable bowel syndrome with mixed diarrhea and constipation, but none had predominantly constipation. However, after 12 weeks constipation was present in 29%, and the rate of mixed diarrhea and constipation had dropped to 14%. The placebo group had no changes in irritable bowel complaints. Although it is important not to over interpret these findings in light of the small sample size, any treatment that would improve the severe gastrointestinal complaints of GWI would be welcomed.

A β-alanine enriched diet has anxietolytic effects in mice (Murakami & Furuse, 2010). Scores on the Generalized Anxiety Disorder 7 (GAD-7) (Löwe et al., 2008) and State-Trait Anxiety Inventory (Spielberger, 1985) questionnaires did not change with carnosine treatment. Anxiety was present in 50% of all CMI subjects based on GAD-7 scores ≥ 10 (Löwe et al., 2008). Depressive affect was inferred at different rates ranging from 81% overall using Center for Epidemiological Studies – Depression (CES-D) scores ≥ 16 (Okun, Stein, Bauman, & Silver, 1996; Radloff, 1977), 49% for Beck Depression Inventory (BDI) scores ≥ 17 (Beck, Steer, Ball, & Ranieri, 1996; Geisser, Roth, & Robinson, 1997), and 36% for major and 21% for minor depressive affects using the PRIME MD questionnaire (Aiarzaguena, Grandes, Salazar, Gaminde, & Sánchez, 2008). These high rates were anticipated based on the severity of the symptom profile of these CMI subjects. These states were resistant to carnosine therapy.

The study was limited by the relatively small sample size. The putative treatment effects require validation in similar randomized double blind placebo-controlled studies with larger groups of participants. The small sample size was hampered even further by the nine subjects who withdrew. They had no apparent symptomatic changes or adverse events. This includes parasthesias that have been noted previously (Artioli et al., 2010).

Study compliance was excellent based on diaries and pill counts. However, there were no cerebrospinal fluid, serum, or urine measurements of carnosine, beta-alanine or histidine to confirm absorption, pharmacodynamics, or brain metabolism to homocarnosine. Elevations in brain GABA levels in the treatment group may be identified by molecular spectroscopy (analysis in progress).

Another limitation was the subjective nature of many of the outcome measures. This was by design for this pilot study to assess a broad set of symptomatic and psychometric variables that may have shown beneficial responses to this antioxidant therapy. For instance, even though we observed a possible significant improvement in symptoms of diarrhea, there are several different sets of criteria for IBS, and this treatment effect could be better confirmed with more precise measures for stool form and frequency. In addition, subjects’ scores for many questionnaire items were at the upper limits, suggesting possible ceiling effects. Small improvements in their conditions may not have been detectable with the scales employed.

Hyperalgesia and disability leading to inactivity were assessed in semi-objective fashion by dolorimetry and actigraphy, respectively. These outcomes were insensitive to change. It is possible that 12 weeks of add-on therapy without adjunctive treatments was insufficient to reverse the consequences of long duration neural dysfunction. In particular, the chronicity of the subjects’ complaints may have made it difficult for them to assess and identify subjective improvements. Established neural patterns may be resistant to modification. Improved understanding of the molecular pathophysiology is required in order to optimally select fMRI and other objective outcome measures for treatment studies such as this one.

## 5. Conclusion

This randomized double blind placebo controlled 12 week dose escalation trial of carnosine showed an increase in digit symbol substitution score and potentially decreased diarrhea in GWI. Similar larger studies with objectively defined and pathophysiologically based outcome measures will be required to confirm and extend these findings.
